# An Engineering Zirconia Ceramic Made of Baddeleyite

**DOI:** 10.3390/ma14164676

**Published:** 2021-08-19

**Authors:** Vyacheslav V. Rodaev, Andrey O. Zhigachev, Alexander I. Tyurin, Svetlana S. Razlivalova, Viktor V. Korenkov, Yuri I. Golovin

**Affiliations:** Institute for Nanotechnology and Nanomaterials, Derzhavin Tambov State University, Internatsionalnaya Str. 33, 392000 Tambov, Russia; andreyzhig2009@yandex.ru (A.O.Z.); tyurin@tsu.tmb.ru (A.I.T.); razlivalova8@yandex.ru (S.S.R.); ya.vikkor@yandex.ru (V.V.K.); golovin@tsu.tmb.ru (Y.I.G.)

**Keywords:** zirconia ceramic, baddeleyite, high-energy milling, phase composition, mechanical characteristics

## Abstract

Wet high-energy milling and uniaxial pressing are used to fabricate CaO-stabilized tetragonal zirconia polycrystalline ceramic (Ca-TZP) with decent mechanical characteristics, i.e., a hardness of 11.5 GPa, Young’s modulus of 230 GPa, and fracture toughness of 13 MPa·m^0.5^. The effect of CaO concentration and the sintering temperature on phase composition and mechanical characteristics of CaO-stabilized zirconia ceramic made of baddeleyite is investigated.

## 1. Introduction

Zirconia can exist in three allotropic forms: monoclinic (m-ZrO_2_), tetragonal (t-ZrO_2_), and cubic (c-ZrO_2_). Pure zirconia is monoclinic from ambient temperature to 1170 °C. In the range of 1170–2370 °C, it is tetragonal, and from 2370 °C to the melting point, it is cubic [1]. Upon cooling reverse t-ZrO_2_→m-ZrO_2_, a transition occurs at 950 °C which is accompanied with an increase in volume by about 4.5% [1]. It leads to unalloyed zirconia cracking upon cooling and provides its poor mechanical properties. Various stabilizing oxides are used to prevent an undesirable t-ZrO_2_→m-ZrO_2_ transition and to keep t-ZrO_2_ at room temperature [2]. Stabilized zirconia, consisting of t-ZrO_2_ grains, are called tetragonal zirconia polycrystals (TZP). TZP exists in the certain range of the dopant concentrations.

TZP ceramics are attractive because of their excellent room-temperature mechanical properties [3]. Mainly TZP ceramics are produced through chemical processing of zircon. Baddeleyite is another natural source of zirconia containing monoclinic ZrO_2_ in the range of 96.5–98.5 wt%. Baddeleyite is much cheaper than zirconia synthesized from zircon, but chemical ways of stabilizing are not efficient for baddeleyite. In [4], high-energy milling was successfully applied to baddeleyite to prepare CaO-stabilized ZrO_2_ nanopowder. CaO was chosen as an inexpensive alternative to Y_2_O_3_ and CeO_2_ which commonly used for producing engineering TZP ceramics.

Mechanical properties of TZP ceramics strongly depend on its grain size [5]. Above a critical grain size, TZP ceramics are susceptible to spontaneous t-ZrO_2_→m-ZrO_2_ transformation throughout the material volume. Below a certain grain size, local t-ZrO_2_→m-ZrO_2_ transformation induced by mechanical impact, giving rise to TZP ceramics toughening, does not occur, resulting in reduced TZP ceramics fracture toughness. The increase in sintering temperature and time leads to larger grain size [5].

The aim of this work is to define CaO concentration and the sintering regime which gives rise to a Ca-TZP ceramic originating from baddeleyite which possesses competitive mechanical properties.

## 2. Materials and Methods

We produced 1–5 wt% CaO-stabilized ZrO_2_ powders by wet high-energy co-milling of CaO powder (Sigma-Aldrich, Saint Louis, Missouri, USA) and the baddeleyite concentrate powder with zirconia content of 99.3% (5 μm, Kovdorsky mining and processing plant, Kovdor, Russia) using the planetary mill Pulverisette 7 Premium Line (Fritsch, Idar-Oberstein, Germany) in the same way as described in [4]. The size of zirconia nanoparticles in the prepared powders is less than 20 nm. We also used the powder of chemically synthesized monoclinic zirconia (99.9%, 5 μm, Sigma-Aldrich, Saint Louis, Missouri, USA) instead of the baddeleyite concentrate to produce reference samples.

The obtained powders were uniaxially pressed under 560 MPa into pellets of a 10 mm diameter and a 2 mm thickness. The fabricated pellets were sintered at the temperature range from 1100 to 1400 °C for 4 h in air atmosphere in a muffle furnace. The sintering temperature was 500 °C and the cooling rate was 5 °C/min. The samples were cooled naturally from 500 °C to room temperature.

Phase composition analysis of sintered ceramics was performed with the help of an X-ray diffractometer (XRD) D2 Phaser (Bruker AXS, Karlsruhe, Germany) at room temperature. The XRD patterns were recorded in the 20–80° 2θ range and assigned using the PDF-2 Diffraction Database File compiled by the International Centre for Diffraction. Phase content was determined from the XRD patterns by the Rietveld method in the TOPAS software (Bruker AXS, Karlsruhe, Germany). The average grain size of t-ZrO_2_ and c-ZrO_2_ was calculated in the TOPAS software (Bruker AXS, Karlsruhe, Germany) by applying the Scherrer equation to the characteristic peaks of the tetragonal and cubic phases located in the 20–80° 2θ range.

Young’s modulus of the samples was measured on a nanoindentometer G200 (MTS Nano Instruments, Oak Ridge, TN, USA) equipped with a Berkovich diamond indenter. Young’s modulus of the samples was calculated from the load-penetration depth curves obtained under peak load of 5 N using the Oliver–Pharr method [6]. Hardness of the samples was measured by Vickers indentation with a load of 19.62 N on a hardness tester Duramin A300 (Struers, Copenhagen, Denmark). Fracture toughness (*K_C_*) was calculated from length of radial cracks starting from the corners of the indents with the Anstis equation [7]:

(1) $$K_{C}=0.016{\left(\frac{E}{H}\right)}^{0.5}\frac{P}{C^{1.5}}$$

where *H* is Vickers hardness, *E* is Young’s modulus, *P* is indentation load to produce cracks, and *C* is the crack length (the distance between the center of the indent and crack tip). The Vickers indentation fracture toughness tests were performed on a hardness tester Duramin A300 (Struers, Copenhagen, Denmark) using loads of 196.2 and 294.3 N. Cracks lengths were calculated using optical microscope Axio Observer.A1m (Carl Zeiss, Oberkochen, Germany). Before tests, all the samples were polished with diamond-containing slurries. All mechanical characteristics measurements were carried out at room temperature. Sintered ceramics containing m-ZrO_2_ are not tested because of numerous cracks already present in the samples before measurements.

## 3. Results and Discussion

**Figure 1:**
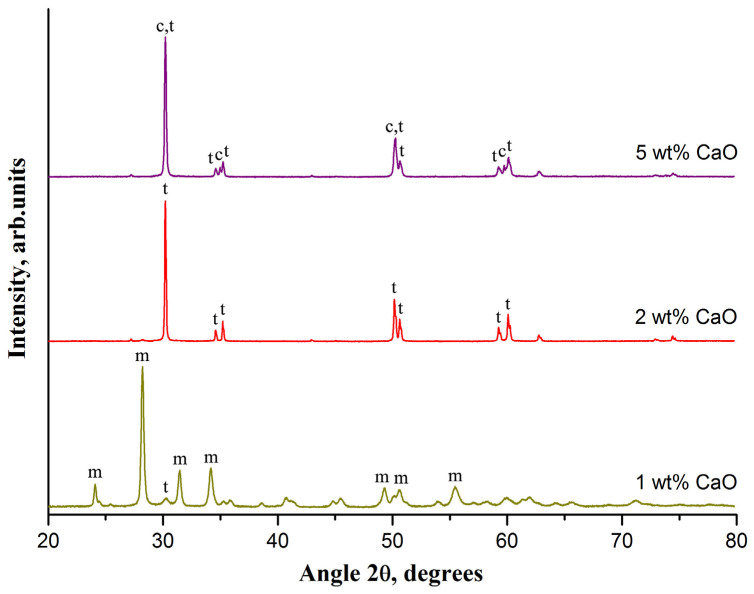
The XRD patterns of a CaO-ZrO_2_ ceramic sintered at 1300 °C containing different dopant amount; m, t, c—the most intense characteristic peaks of m-ZrO_2_, t-ZrO_2_ and c-ZrO_2_, respectively.

Figure 1 shows ceramics XRD patterns evolution with the rise in the dopant CaO concentration increase at a fixed sintering temperature of 1300 °C.

The m-ZrO_2_ phase dominates in the ceramic if 1-wt% CaO is used. The characteristic peaks of m-ZrO_2_ at 24.1°, 28.2°, 31.5°, 34.2°, 49.3°, 50.6°, and 55.5° are observed in the XRD pattern. The most intensive characteristic peak of t-ZrO_2_ at 30.2° is very weak compared to m-ZrO_2_ reflections. Phase composition of ceramic dramatically changes at 2 wt% CaO. The peaks of m-ZrO_2_ disappear and only the peaks of t-ZrO_2_ at 30.2°, 34.6°, 35.2°, 50.2°, 50.7°, 59.3°, and 60.2° are observed in the XRD pattern. This indicates that a 2-wt% CaO-ZrO_2_ ceramic is a TZP ceramic. A further increase in CaO concentration from 3 to 5 wt% leads to an increase in c-ZrO_2_ content from 7 to 23 wt% and, respectively, t-ZrO_2_ content reduction from 93 to 77 wt%. The intensity of the c-ZrO_2_ peaks increase with the rise in c-ZrO_2_ content. The characteristic peaks of c-ZrO_2_ at 35.0° and 59.7° are clearly observed in the XRD pattern of a 5-wt% CaO-ZrO_2_ ceramic. Other characteristic peaks of c-ZrO_2_ at 30.1° and 50.2° overlap with the neighboring peaks of t-ZrO_2_. With the rise in CaO concentration from 2 to 5 wt%, the average grain size of t-ZrO_2_ decreases from 93 to 63 nm and the average grain size of c-ZrO_2_, on the contrary, increases to 83 nm.

**Figure 2:**
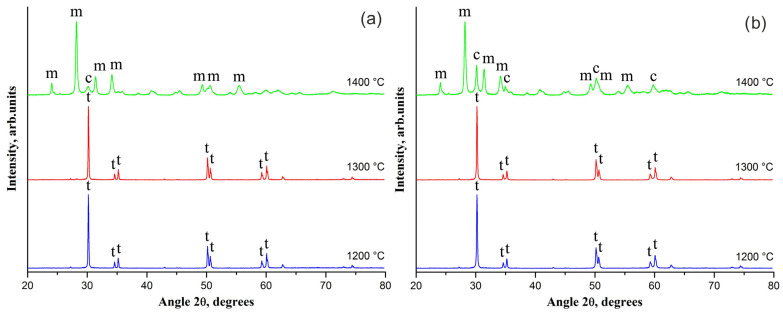
The XRD patterns of (**a**) 2-wt% CaO-ZrO_2_ and (**b**) 3-wt% CaO-ZrO_2_ ceramics sintered at temperatures in the range of 1200–1400 °C; m, t, c—the most intense characteristic peaks of m-ZrO_2_, t-ZrO_2_ and c-ZrO_2_, respectively.

It is revealed that phase composition of CaO-ZrO_2_ ceramic is sensitive to the sintering temperature higher than 1300 °C despite CaO concentration (Figure 2). If the sintering temperature is increased to 1400 °C m-ZrO_2_ becomes to dominate. The t-ZrO_2_ is practically absent in a ceramic and c-ZrO_2_ content increases with the rise in CaO concentration as evidenced by an increase in the intensity of the characteristic peaks of c-ZrO_2_.

**Figure 3:**
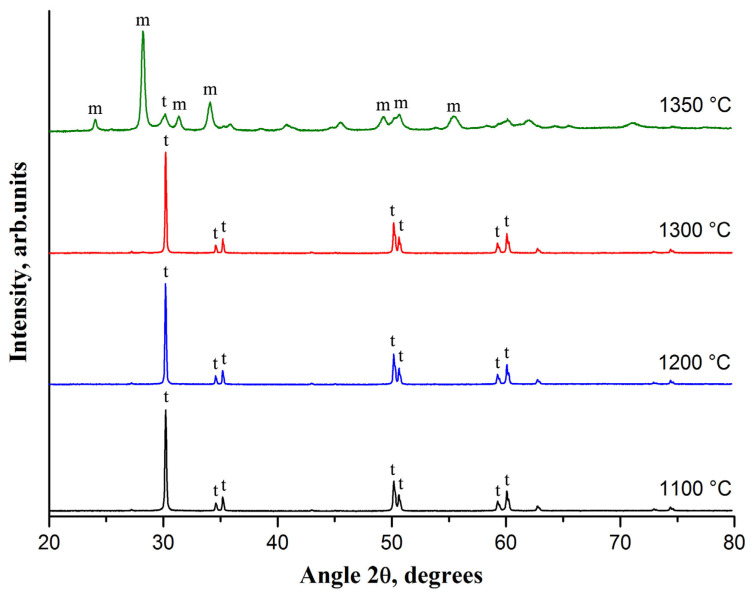
The XRD patterns of 2-wt% CaO-ZrO_2_ ceramic sintered at different temperatures; m, t—the most intense characteristic peaks of m-ZrO_2_ and t-ZrO_2_, respectively.

Figure 3 presents the effect of the sintering temperature on phase composition of the fabricated TZP ceramic doped by 2-wt% CaO.

It is found that the 2-wt% CaO-ZrO_2_ ceramic consists only of t-ZrO_2_ if sintering is performed at 1300 °C and lower temperatures. An increase in the sintering temperature induces zirconia grains growth. The average grain size of t-ZrO_2_ increases from 79 to 93 nm with the rise in the sintering temperature from 1100 to 1300 °C. Phase composition of the 2-wt% CaO-ZrO_2_ ceramic dramatically changes if sintering temperature of 1350 °C is used. The TZP ceramic transforms into one mainly composes of m-ZrO_2_. The radical change in phase composition of the 2-wt% CaO-ZrO_2_ ceramic with the rise in the sintering temperature from 1300 to 1350 °C is related to critical t-ZrO_2_ grain size. In [8], it was found that, in a Ca-TZP ceramic, the t-ZrO_2_ grain size cannot exceed 100 nm. The Ca-TZP ceramic examined in [8] was produced using uniaxial pressing from a nanopowder obtained by hydrothermal treatment of the co-precipitated calcium and zirconium hydroxides. In our case, the average grain size of t-ZrO_2_ is 93 nm if the Ca-TZP ceramic is sintered at 1300 °C. For comparison, in Y_2_O_3_-stabilized TZP ceramics, t-ZrO_2_ grains up to 1 μm in size can exist [9]. It explains higher sintering temperatures applying to Y_2_O_3_-stabilized TZP ceramics.

Peaks’ broadening in the XRD patterns of m-ZrO_2_ containing ceramics (Figure 1, Figure 2 and Figure 3) may be due to significant mechanical stresses resulting in visually observed ceramic cracking.

Mechanical characteristics of CaO-containing zirconia ceramics made from baddeleyite were examined. It was found that an increase in CaO concentration from 2 to 5 wt% leads to a rise in hardness by 5.5% and a decrease in fracture toughness by 29.9% (Table 1). Wherein, Young’s modulus remains unchanged within the measurement error.

It can be seen from Table 1 that a 2-wt% CaO-ZrO_2_ ceramic being TZP ceramic has the best combination of mechanical characteristics and the highest fracture toughness value. In terms of hardness and Young’s modulus, the fabricated 2-wt% CaO-ZrO_2_ ceramic corresponds to engineering Y-TZP ceramics made of chemically synthesized zirconia stabilized with Y_2_O_3_, and surpasses them in terms of fracture toughness [1]. High fracture toughness of TZP ceramics is a result of transformation toughening [10]. The stress field in the crack tip zone induces local t-ZrO_2_→m-ZrO_2_ transformation which causes volume expansion and shear strains. It applies compressive stress at the crack tip to prevent crack propagation. As a result, further crack growth is suppressed and strength of a ceramic increases. The rise in a dopant concentration leads to a decrease in the fraction of transformable t-ZrO_2_ in favor of c-ZrO_2_ incapable of transformation. It explains fracture toughness reduction in the fabricated zirconia ceramic with an increase in CaO concentration (Table 1). It can be seen that ceramic hardness increases when fracture toughness decreases. We suppose that it is due to lower hardness of the m-ZrO_2_ ceramic compared to the t-ZrO_2_ one. Indeed, hardness measurements induce t-ZrO_2_→m-ZrO_2_ transformation under an indenter tip. Thus, resulting hardness includes hardness of t-ZrO_2_ and m-ZrO_2_ phases. A decrease in the fraction of transformable t-ZrO_2_ negatively affects mechanically-induced t-ZrO_2_→m-ZrO_2_ transformation and respectively reduces m-ZrO_2_ contribution to the resulting hardness. To confirm this assumption, we tested a 1-wt% CaO-ZrO_2_ ceramic, containing more than 90 wt% m-ZrO_2_, using a nanoindentometer due to numerous cracks in the sample. Hardness was calculated from the obtained load–penetration depth curves using the Oliver–Pharr method. The prints size was significantly smaller than the analyzed region bounded by the cracks. A 5-wt% CaO-ZrO_2_ ceramic, mainly composed of t-ZrO_2_ with a reduced ability to t-ZrO_2_→m-ZrO_2_ transformation, was tested on a nanoindentometer too. A nanoindentation by a Berkovich diamond indenter with a tip of a 20-nm radius showed that hardness of a 1-wt% CaO-ZrO_2_ ceramic is significantly lower than the hardness of a 5-wt% CaO-ZrO_2_ ceramic, namely, 8.24 ± 0.37 GPa versus 12.63 ± 0.41 GPa.

It should be noted that an increase in CaO-ZrO_2_ ceramic hardness with the rise in CaO concentration cannot be explained in terms of the empirical Hall-Petch relationship [11], which describes the phenomenon whereby hardness (or strength) of materials increases with reducing the grain size:

(2) $$H=H_{0}+\frac{k}{\sqrt{D}}$$

where *H* is the measured hardness, *H*_0_ is the intrinsic hardness related to the resistance of lattice to dislocation motion, *k* is the material-specific strengthening coefficient, and *D* is the average grain size.

**Figure 4:**
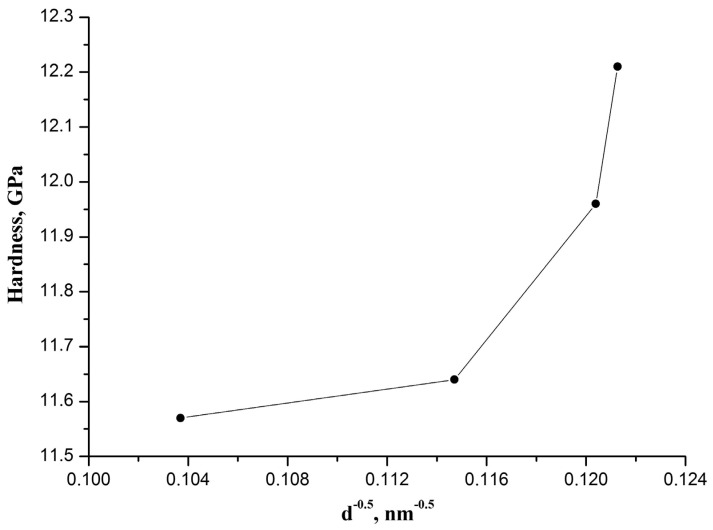
The dependence of hardness of the CaO-ZrO_2_ ceramic sintered at 1300 °C containing a different CaO amount on the inversed square root of the effective grain size. The dots are connected by lines for clarity.

The observed dependence of CaO-ZrO_2_ ceramic hardness on the inversed square root of the effective grain size is not a line as required by the Hall–Petch relationship (Figure 4). The effective grain size of the CaO-ZrO_2_ ceramic was calculated for each CaO concentration from the range of 2–5 wt% knowing the content of t-ZrO_2_ and c-ZrO_2_ as well as the average grain size of t-ZrO_2_ and c-ZrO_2_ (Table 2).

It is revealed that the rise in the sintering temperature in the range of 1100–1200 °C results in simultaneous increase in hardness, fracture toughness, and Young’s modulus of a 2-wt% CaO-ZrO_2_ ceramic (Table 3) despite its phase composition constancy (Figure 3). It allows concluding that the observed improvement in mechanical properties is related to ceramic densification.

Indeed, the sintering temperature of 1100 °C is lower than the Tammann temperature of zirconia [12]; therefore, the fabricated ceramic is unsintered and possesses poor mechanical characteristics. On the contrary, if sintering occurs at 1200–1300 °C, a well-sintered dense ceramic with decent mechanical characteristics is obtained. The density of a 2-wt% CaO-ZrO_2_ ceramic sintered at temperatures of 1100–1300 °C measured by the Archimedes method with distilled water as the immersing medium is presented in Table 4.

According to the data in Table 3 and Table 4 the rise in the sintering temperature leads to the ceramic densification (porosity reduction), which improves the mechanical characteristics of the ceramic. The similar effect was observed, for example, in [13] where an increase in the sintering temperature resulted in Y_2_O_3_-stabilized TZP ceramic densification, which in turn led to the rise in Young’s modulus, bending strength and fracture toughness of the given ceramic.

Fracture toughness of a 2-wt% CaO-ZrO_2_ ceramic is 35.5% higher if sintering occurs at 1300 °C and not at 1200 °C. It can be related to the over-stabilization effect [2] since phase composition of the ceramic remains unchanged with the rise in the sintering temperature from 1200 to 1300 °C. For a given dopant concentration, TZP ceramic fracture toughness decreases as t-ZrO_2_ grains become smaller and their ability to t-ZrO_2_→m-ZrO_2_ transformation reduces. An increase in the sintering temperature induces observed t-ZrO_2_ grains growth and thus facilitates t-ZrO_2_→m-ZrO_2_ transformation following the introduction of a crack.

To reduce energy consumption, the effect of sintering time on mechanical characteristics of a 2-wt% CaO-ZrO_2_ ceramic was examined too. It was found that sintering time reduction from 4 to 1 h has no effect on hardness, fracture toughness, and Young’s modulus of a 2-wt% CaO-ZrO_2_ ceramic within the measurements error. The 2-wt% CaO-ZrO_2_ ceramic sintered at 1300 °C for 1 h is characterized by the following mechanical characteristics: hardness of 11.52 ± 0.05 GPa, Young’s modulus of 226 ± 8 GPa, and fracture toughness of 12.83 ± 0.41 MPa·m^0.5^.

The used baddeleyite concentrate contains impurities in the amount of 0.7 wt%. The dominating ones are SiO_2_, SO_3_, P_2_O_5_, TiO_2_, Fe_2_O_3_, CaO, and MgO [14]. To investigate the effect of impurities on the mechanical characteristics of zirconia ceramics, we compared hardness, fracture toughness, and Young’s modulus of CaO-ZrO_2_ ceramics made of the baddeleite concentrate and chemically synthesized monoclinic zirconia. According to Table 1, 2-wt% CaO-ZrO_2_ ceramics sintered at 1300 °C were chosen for testing. It was found that both ceramics made of chemically synthesized monoclinic zirconia and the baddeleyite concentrate are TZP ceramics. Their mechanical characteristics are given in Table 5.

**Figure 5:**
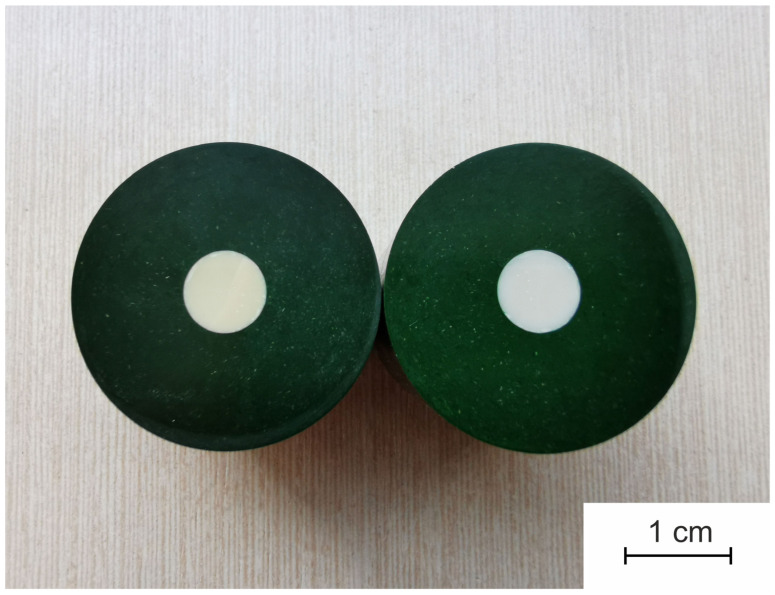
The photo of 2-wt% CaO-ZrO_2_ ceramics made of the baddeleyite concentrate (**left**) and chemically synthesized monoclinic zirconia (**right**). The samples are prepared for mechanical tests.

It can be seen from Table 5 that the values of analyzed mechanical characteristics for both ceramics are similar. It indicates that impurities amount in the baddeleyite concentrate is too low to worsen the mechanical characteristics of resulting ceramics. Thus, zirconia ceramics with the same mechanical characteristics can be produced using the baddeleyite concentrate as a raw material instead of the more expensive chemically synthesized monoclinic zirconia. However, it should be noted that zirconia ceramic made of the baddeleyite concentrate is yellow-tinted due to impurities (Figure 5).

## 4. Conclusions

Optimal dopant concentration and the sintering regime to obtain Ca-TZP ceramic originating from baddeleyite with competitive mechanical characteristics are defined. The 2-wt% CaO-ZrO_2_ ceramic sintered at 1300 °C for 1 h possesses hardness ~11.5 GPa, Young’s modulus ~230 GPa, and fracture toughness ~13 MPa·m^0.5^. In terms of hardness and Young’s modulus, the fabricated 2-wt% CaO-ZrO_2_ ceramic corresponds to engineering Y-TZP ceramics made of chemically synthesized zirconia stabilized with Y_2_O_3_, and surpasses them in terms of fracture toughness. It is revealed that the rise in the CaO concentration from 2 to 5 wt% stimulates c-ZrO_2_ content increase and a Ca-TZP ceramic transforms into a ceramic, consisting of both t-ZrO_2_ and c-ZrO_2_. An increase in CaO concentration leads to the rise in hardness and a decrease in fracture toughness of a CaO-ZrO_2_ ceramic wherein its Young’s modulus remains unchanged. Furthermore, it is found that Ca-TZP ceramic cannot be produced if the sintering temperature of 1350 °C or higher is used.

## Figures and Tables

**Table 1:** Mechanical characteristics of CaO-stabilized zirconia ceramics of a baddeleyite origin sintered at 1300 °C containing different dopant amount.

CaO Concentration, wt%	Hardness, GPa	Fracture Toughness, MPa·m^0.5^	Young’s Modulus, GPa
2	11.57 ± 0.10	13.14 ± 0.49	228 ± 10
3	11.64 ± 0.06	11.89 ± 0.27	227 ± 5
4	11.96 ± 0.09	10.44 ± 0.41	223 ± 6
5	12.21 ± 0.06	9.21 ± 0.35	225 ± 9

**Table 2:** The content of t-ZrO_2_ and c-ZrO_2_ phases and the average grain size of t-ZrO_2_ and c-ZrO_2_ phases in CaO-ZrO_2_ ceramic sintered at 1300 °C containing a different CaO amount.

CaO Concentration, wt%	t-ZrO_2_		c-ZrO_2_	
	Content, wt%	Average Grain Size, nm	Content, wt%	Average Grain Size, nm
2	99	93	1	-
3	93	78	7	57
4	86	69	14	67
5	77	63	23	83

**Table 3:** Mechanical characteristics of a 2-wt% CaO-ZrO_2_ ceramic of a baddeleyite origin sintered at different temperatures.

Sintering Temperature, °C	Hardness, GPa	Fracture Toughness, MPa·m^0.5^	Young’s Modulus, GPa
1100	6.34 ± 0.09	4.96 ± 0.39	131 ± 11
1200	11.93 ± 0.07	9.70 ± 0.34	227 ± 11
1300	11.57 ± 0.10	13.14 ± 0.49	228 ± 10

**Table 4:** The density and the relative density of 2 wt% CaO-ZrO_2_ ceramic of a baddeleyite origin sintered at different temperatures.

Sintering Temperature, °C	Density, cm^3^/g	Relative Density, %
1100	4.37	72.8
1200	5.85	97.5
1300	5.96	99.3

**Table 5:** Mechanical characteristics of 2-wt% CaO-ZrO_2_ ceramics made of the baddeleyite concentrate and chemically synthesized monoclinic zirconia. The sintering temperature is 1300 °C.

Raw Material of 2 wt% CaO-ZrO_2_ Ceramic	Hardness, GPa	Fracture Toughness, MPa·m^0.5^	Young’s Modulus, GPa
Baddeleite concentrate	11.57 ± 0.10	13.14 ± 0.49	228 ± 10
Chemically synthesized monoclinic zirconia	11.97 ± 0.12	12.86 ± 0.43	221 ± 11

## Data Availability

All data included in this study are available upon request from the corresponding author.
